# Report on noninvasive prenatal testing: classical and alternative approaches

**DOI:** 10.12688/f1000research.8243.1

**Published:** 2016-04-22

**Authors:** Kateryna S. Pantiukh, Nikolay N. Chekanov, Igor V. Zaigrin, Alexei M. Zotov, Alexander M. Mazur, Egor B. Prokhortchouk

**Affiliations:** 1 Genoanalytica, CJSC, Moscow, Russian Federation; 2 Faculty of Physics, Moscow State University, Moscow, Russian Federation; 3 Research Center of Biotechnology RAS, Moscow, Russian Federation; 4 Faculty of Biology, Moscow State University, Moscow, Russian Federation

**Keywords:** noninvasive prenatal testing (NIPT), trisomy, aneuploidy, cell-free DNA, fetal cell-free DNA concentration, Down syndrome, Edwards syndrome, Patau syndrome

## Abstract

Concerns of traditional prenatal aneuploidy testing methods, such as low accuracy of noninvasive and health risks associated with invasive procedures, were overcome with the introduction of novel noninvasive methods based on genetics (NIPT). These were rapidly adopted into clinical practice in many countries after a series of successful trials of various independent submethods.

Here we present results of own NIPT trial carried out in Moscow, Russia. 1012 samples were subjected to the method aimed at measuring chromosome coverage by massive parallel sequencing. Two alternative approaches are ascertained: one based on maternal/fetal differential methylation and another based on allelic difference. While the former failed to provide stable results, the latter was found to be promising and worthy of conducting a large-scale trial.

One critical point in any NIPT approach is the determination of fetal cell-free DNA fraction, which dictates the reliability of obtained results for a given sample. We show that two different chromosome Y representation measures—by real-time PCR and by whole-genome massive parallel sequencing—are practically interchangeable (r=0.94). We also propose a novel method based on maternal/fetal allelic difference which is applicable in pregnancies with fetuses of either sex. Even in its pilot form it correlates well with chromosome Y coverage estimates (r=0.74) and can be further improved by increasing the number of polymorphisms.

## Introduction

Aneuploidies can be attributed to cause 30% of miscarriage cases, and affect up to 1 in 300 live births ^1^. Most common autosomal aneuploidies are the trisomies of 21st, 18th and 13th chromosomes ^1^. While still causing various health defects and intellectual disabilities, normally they are not lethal to fetus, in contrast to many other chromosomal abnormalities.

Recently a new noninvasive prenatal testing technology based on sequencing of cell-free DNA from maternal blood was widely implemented in the industry. Blood plasma of a pregnant woman contains cell-free DNA fragments of both maternal and fetal origin. The latter permeates through the placental barrier into the main blood flow. Fetal cfDNA (cffDNA) emerges during the apoptosis of cytotrophoblast cells ^2^. Fetal fraction makes up 10–20% of all blood plasma cfDNA on average, rising through the whole pregnancy duration. After labor it disappears from the blood flow in several hours ^3^. It was shown that fragments of cffDNA uniformly represent the whole genome of the fetus ^4^. Trisomy of a certain chromosome in the fetus may be detected through sequencing of total cfDNA from maternal blood plasma and subsequent counting of reads mapped on each chromosome. Such chromosome would show statistically significant increase in coverage ^5– 7^. In 2% of cases at 10th through 21st weeks of gestation, cffDNA fraction comprises less than 4% ^8^. Underrepresentation of fetal genetic material might lead to false negative outcomes, so such cases must be diagnosed by other means.

Sequencing of cell-free DNA from maternal blood proved to be a technique which is completely safe, highly accurate, and shows high potential for extendability. Since its introduction into clinical practice in 2011, it has spread quickly and now is available in most developed countries. Its precision was confirmed in a number of studies on hundreds of thousands of samples combined, showing accuracy rates of more than 99%, which is especially intriguing given a wide variety of statistical methods employed by different providers. Here we describe our experience with introduction of NIPT in Russia. The test was developed at the Genoanalytica private laboratory.

We chose whole-genome low coverage sequencing with GC correction ^9^ as our main method. It doesn't rely on prior knowledge of population and yields stable and reproducible results. 1012 samples were analyzed with the main method to assess its performance. Two additional methods for aneuploidy detection were evaluated: one based on differential DNA methylation between mother and fetus, and another based on difference in allele content.

Determination of the cffDNA fraction is a crucial part of the NIPT pipeline, as samples with low concentration must be treated with caution. We addressed this issue in the second part of this article. Several techniques for calculation of cffDNA percentage were employed: 1) a method based on RT-PCR detection of sequences coming from Y-chromosome in samples with male fetus; 2) a method based on counting sequenced reads mapped on Y-chromosome in samples with male fetus; 3) a method based on deep sequencing of several highly polymorphic regions to assess differences in allele content between mother and fetus; and 4) a method based on sequencing of polymorphisms with an additional stage for eliminating PCR duplicates. It's worth mentioning that the two latter methods are self-reliant and do not require prior genotyping of either parent.

## Methods

### Aneuploidy detection

***Patients and sample collection.*** The study design was approved by the Institutional Review Board of Genoanalytica, CJSC, approval no. 103/2015.

A total of 1012 women participated in the study. All participants gave informed consent for providing their blood samples for scientific purposes. The cohort included cases with both high and low risk of aneuploidies as typical screening procedures suggested. Biochemical or ultrasonographic markers, as well as advanced age (35 years and older) were considered as high risk factors. A karyotyping report for the fetus, or postnatal diagnosis were obtained in all studied pregnancies.

Blood samples were transported to the laboratory in tubes with EDTA at 4°C no longer than 4 hours after drawing. Whole blood was centrifuged for 10 minutes at 1600g twice sequentially. After that plasma was separated and placed in new tubes, which were again centrifuged for 10 minutes at 1600g and then stored at 4°C. Cell-free DNA was extracted from stored blood plasma using QIAamp DNA Circulating Nucleic Acid Kit (Qiagen) according to the manufacturer’s instructions. The amount of DNA was determined on Qubit 2.0 (Invitrogen) using Qubit dsDNA HS Assay Kit (Invitrogen) according to the manufacturer’s instructions.

## Sequencing, bioinformatics analysis and confirmation of results

### Aneuploidy detection (whole-genome low coverage)

Whole-genome libraries were prepared according to standard Illumina protocol. Their quality was assessed with Bioanalyzer using High Sensitivity DNA Analysis Kits (Agilent Technologies). 5 to 10 million reads of 50bp length were obtained on HiSeq 1500 (Illumina) for every sample. Sequencing data were processed as in 9, and cffDNA concentration calculated according to 10.

After the test every participant was consulted according to her results. Invasive diagnostic procedure (karyotyping via amniocentesis or similar measure) was suggested to women with positive test results; those denying to do so were monitored until the birth to obtain the medical outcome data. For participants with negative test results outcome data was obtained through telephone interview a month after the expected date of birth.

### Aneuploidy detection (differential methylation)

6 blood samples from pregnant women were selected from the main cohort to test the alternative method employing differential DNA methylation. All of them were at gestational age of 12 weeks; 3 were known to be chromosome 21 trisomic (cytogenetically confirmed) and 3 were known to be normal (confirmed after the birth). One additional sample was obtained from a non-pregnant woman.

16 genomic regions known to be differentially methylated between fetal tissues and maternal blood cells ^11^ were assessed: 12 from chromosome 21 and 4 from other chromosomes. Extracted cfDNA underwent bisulphite conversion using Zymo Research EZ DNA Methylation Kit according to its protocol. Genomic libraries were sequenced on Ion Torrent PGM (Thermo Fisher Scientific) using the 316 chip type.

Acquired reads were subjected to filtering by quality (at least having average of Q20) and length (at least having length of 100bp) and mapped to DMRs. Bisulphite conversion percentage and methylation status at the first two CpG positions were then calculated for every DMR.

### Aneuploidy detection (fetal and maternal polymorphisms)

4 blood samples from pregnant women were selected from the main cohort to test the alternative method based on differences in genotypes of mother and fetus. 2 were known to be chromosome 21 trisomic (cytogenetically confirmed) and 2 were known to be normal (confirmed after the birth). Genome libraries were prepared according to the Ovation Custom Target Enrichment System protocol (NuGen) ^12^. One distinguishing feature of this method is a ligation of random adapter sequence to cfDNA fragments before the amplification. It then can be used to precisely remove PCR duplicates, which otherwise are capable of introducing unwanted allele bias. Sequencing was carried out on a HiSeq 1500 (Illumina). Reads were mapped on reference genome hg19 with bowtie2 and then PCR-duplicates were filtered out using NuGen Ovation Target Enrichment System Data Processing Application ver.1.0.0 (NuGen). SNP-calling was performed with samtools ver.1.1 mpileup. Polymorphisms were classified into either of two groups: homozygous in mother (prevalent maternal allele and minor fetal allele) and heterozygous in mother (no prevalence of either maternal allele with possible slight bias towards one of fetal alleles). Dividing coefficient *a* was calculated with formula ( 1):

*a* = R _min_/R _maj_ (1)

Concentration of cffDNA was predicted for every SNP. One of the formulas ( 2, 3) was applied depending on the type of polymorphism.

           if a > 0.25, C = (R _maj_ – R _min_)/R _sum_ (2)

           if а < 0.25, C = 2 * R _min_/R _sum_ (3)

All such predictions were combined in two distributions: one based on polymorphisms of chromosome 21 only, and another for all other autosomess (except for 13 and 18 chromosomes). These two distributions were then compared using t-test. Samples with p-value < 0.05 were considered to possess a high risk of aneuploidy. These analyses were performed in R version 3.2. 

## Determination of cffDNA concentration

### Comparison of methods for cffDNA concentration determination based on NGS and real-time PCR

All samples from the main cohort were subjected to a sex determination procedure which also included determination of cffDNA concentration for samples with male fetus. These steps were performed using the method of Jiang *et al.* ^13^ which employs calculation of chromosome Y coverage with correction of GC-content bias. To evaluate accuracy of this approach cffDNA concentration was also determined using an alternative method based on real-time PCR ^14^ in 10 samples.

### Deep sequencing of highly polymorphic regions

15 samples in which cffDNA concentration was determined were selected from the main cohort. They underwent target amplification of highly polymorphic genome regions and then proceeded to standard preparation of sequencing libraries. These regions were defined to maximize the number of SNPs with high MAF (0.3–0.5) in a 200bp span. The target panel included 36 such regions with 220 SNPs in total. SNP coordinates and MAF values were obtained from dbSNP v141 and ExAC databases ^15^. Sequencing was carried out on HiSeq 1500 (Illumina). Each sample yielded 1 to 3 mln reads of 150bp length.

Another 10 samples from the main cohort were subjected to similar procedures, with a sonication step added before the preparation of genomic libraries. These were sequenced on the same machine, yielding 3 to 5 mln reads of 50bp length per sample. Reads were mapped to reference sequences of regions (which were extracted from human reference genome UCSC hg19) using the bowtie2 ver.2.2.2 software. SNP calling was performed with the samtools ver.1.1 program (mpileup).

All found polymorphisms were annotated with the following metrics: overall number of reads mapped at the position (raw coverage), number of reads supporting non-reference allele at the position (minor allele coverage), and percentage of reads supporting non-reference allele (minor allele coverage/raw coverage). These percentages were split into two groups (high-MAF with percentages ranging from 35% to 65%, and low-MAF for others). A set of theoretical distributions was prepared to compare with the distribution of high-MAF percentages. It included a normal distribution with a peak at 50% (representing the case with zero percentage of cffDNA), and a sum of two normal distributions with peaks at 50% and 50±1/2x% (x representing the concentration of cffDNA) for every x in range from 1 to 25. The one theoretical distribution which correlates best with the observed distribution would yield the percentage of cffDNA.

### Deep sequencing of highly polymorphic regions excluding PCR duplicates

Sample selection, experimental procedures and calculation details are described in the respective section of methods for aneuploidy detection. Contrary to the aneuploidy detection method, here percentage of cffDNA was calculated not for every chromosome separately, but for all autosomes combined (save for 13, 18 and 21, as these are susceptible to trisomies). Resulting cffDNA percentage was calculated as average of cffDNA concentration values in all polymorphisms. These analyses were performed in R version 3.2.

## Results

Raw data for 'Report on noninvasive prenatal testing: classical and alternative approaches’, Pantiukh et al. 2015 Click here for additional data file. Copyright: © 2016 Pantiukh KS et al. 2016 Data associated with the article are available under the terms of the Creative Commons Zero "No rights reserved" data waiver (CC0 1.0 Public domain dedication).

## Aneuploidy detection (whole genome low-coverage)

### Study cohort

1012 blood samples of pregnant women were collected in a period from April 2014 through April 2015. Patients came from 47 medical institutions, as well as independently, across 16 federal subjects of Russia. The average age of participants was 35 years; the youngest was 20, and the oldest 51. Gestation ages were in the range of 10 to 24 weeks, with a median of 14. The average concentration of cffDNA was 11%.

### NIPT positive and negative cases

**Figure 1.**
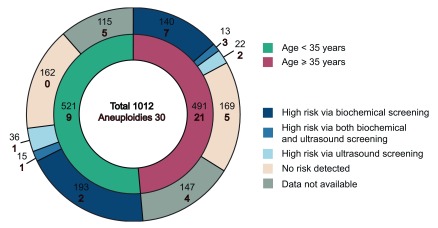
Characterization of sample pool.

For 30 out of 1012 samples (2.9%) a high risk of either aneuploidy was predicted: 25 were T21, 4 T18 and 1 T13. Of these, 24 cases were confirmed via karyotyping (22 T21 and 3 T18), and 3 failed such confirmation (1 T21, 1 T18, 1 T13). 1 other case of predicted T21 was confirmed after childbirth via standard medical examination of the newborn. 2 cases of predicted T21 could not be confirmed due to loss of contact with the participant. Figure 1 describes different indications and outcomes in 30 cases of detected aneuploidies.

A negative result (low risk of either aneuploidy) was returned in 982 cases. Of these, in 813 cases (82.8%) pregnancy ended with labor and thus confirmation was obtained. One case was a false-negative.

Method accuracy metrics are presented in the Table 1. Some of the metrics there are affected by small sample sizes, and will be reevaluated later as the number of participants grows.

**Table 1.** Accuracy metrics of the whole genome low-coverage method.

Aneuploidy	Sensitivity	Specificity	NPV	PPV
T21	95.8%	99.9%	100%	96%
T18	100%	99.9%	100%	80%

With regards to trisomies of chromosome 13, only one case of elevated risk was encountered. This particular result failed to replicate through karyotyping though. The precision of T13 detection with the method was not calculated due to the small sample size.

### Aneuploidy detection (differential DNA methylation)

**Figure 2.**
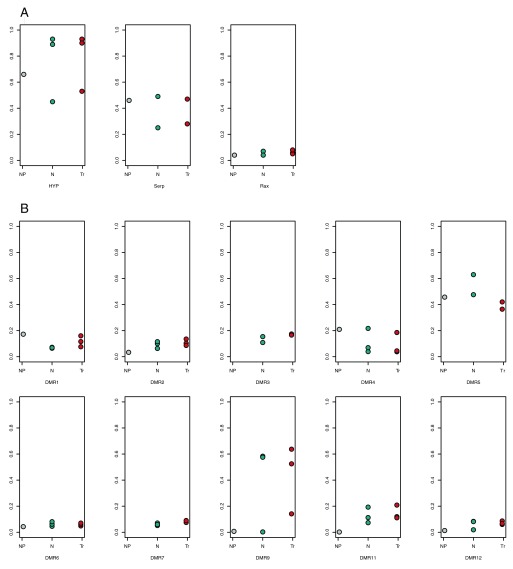
Methylation levels at selected differentially methylated regions (DMRs) in different sample groups. Non-pregnant samples are in gray, samples with normal fetus are in teal, and samples with chromosome 21 trisomic fetus are in red. **A**: DMRs of control chromosomes. **B**: DMRs of chromosome 21.

Here we will define DMRs as regions of genome highly methylated in DNA of fetal origin, but with low methylation in maternal DNA. Concentration of cffDNA thus could be assessed as the percentage of methylated reads obtained by cell-free DNA sequencing targeted at these particular DMRs. Levels of methylation were determined separately for DMRs on chromosome 21 and for control DMRs coming from all other autosomes save for chromosomes 13 and 18. Three groups of samples were considered: pregnant women (fetus with normal karyotype), pregnant women (fetus with T21) and one non-pregnant woman. Both chromosome 21 and control DMRs showed high within-group variance of methylation ( Figure 2). Methylation of control DMRs was unexpectedly high and did not differ significantly when compared between pregnant and non-pregnant women. Only the 2nd and 9th DMRs of chromosome 21 clearly indicated such difference. We expected methylation levels to rise in chromosome 21-trisomic samples compared to normal ones when considering DMRs of chromosome 21, while DMRs of control chromosomes would show no such change. However, in most cases there was no significant change, except for DMR7. DMR3 also exhibited it, but variation of methylation levels in normal samples made it insignificant.

High within-group variation may be explained either by individual differences or by heterogeneity of sources of cffDNA. CffDNA mainly originates from the cells of placenta and chorionic cilia, so the observed pattern of DNA methylation might be biased by interference of different epigenetic profiles.

Negative factors such as individual differences and nonsignificant maternal-fetal methylation ratios prevent this method from immediate application in clinical NIPT. These may be alleviated through introduction of more suitable DMRs.

### Aneuploidy detection (fetal and maternal polymorphisms)

**Figure 3.**
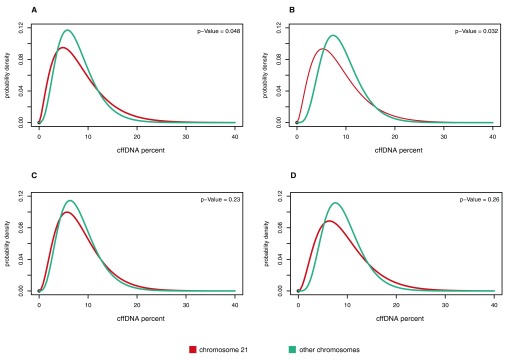
Distributions of polymorphism cffDNA fractions in different samples. Fractions calculated for polymorphisms of chromosome 21 are in red, control chromosomes are in teal. **A** and **B**: samples with chromosome 21 trisomic fetus. **C** and **D**: samples with normal fetus.

This method is based on estimating cffDNA fractions for SNPs in every autosome separately and measuring shift of their distribution in the chromosome of interest. In this study we tested the method on 10 samples (5 normal and 5 with trisomy of chromosome 21). Figure 3 provides examples of these distributions.

Shift of cffDNA fractions' distribution was assessed with t-test; p-value of 0.05 was enough to correctly discern normal samples from problematic ones. However it is worth mentioning that p-values between these series were only one order of magnitude apart, which may point at the risk of low confidence and beg for further inquiry.

## Calculation of cffDNA fraction

### Comparison of methods for cffDNA concentration estimation based on NGS and real-time PCR

**Figure 4.**
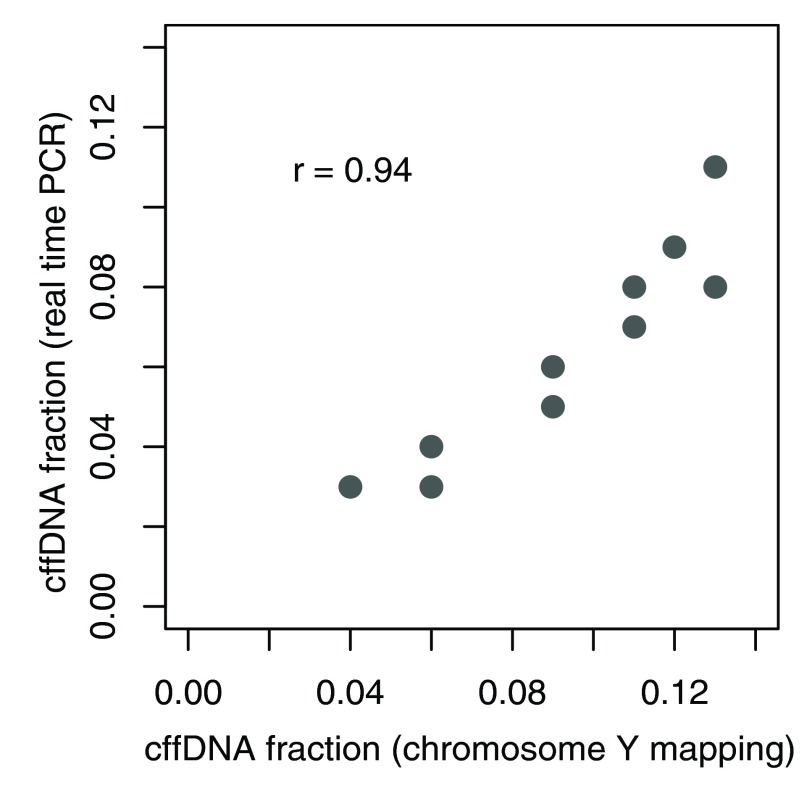
Results of cffDNA fraction estimation based on NGS and real-time PCR.

We have discovered a very high correlation between cffDNA concentration predictions (r=0.94) obtained from real-time PCR and NGS-based chromosome Y presence detection ( Figure 4). We thus conclude that results of both methods closely reflect the real fraction.

As NGS-based method doesn't require any additional experimental procedures we selected it as the reference to compare proposed alternative methods to.

### cffDNA fraction estimation (deep sequencing of high-MAF polymorphisms)

**Figure 5.**
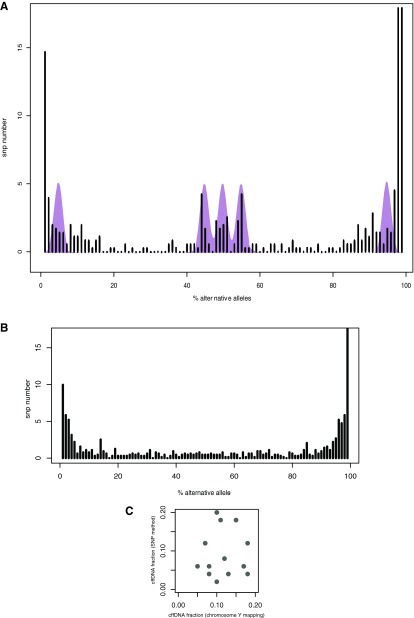
Results of cffDNA fraction estimation based on deep sequencing of high-MAF polymorphisms. **A** and **B**: distribution of allele frequencies of polymorphisms. Theoretical distribution for 12% cffDNA fraction shown in purple. **C**: Results of cffDNA fraction estimation based on allele frequencies of polymorphisms and NGS method.

We have determined allele frequencies of polymorphisms and displayed them in the form of distributions. Figure 5.A shows an example of such distribution in comparison with theoretically expected distribution for a sample with a given cffDNA fraction. 3 peaks can be seen near MAF of 50%. These are SNPs in following maternal-fetal genotype configurations: AB _m_AB _f_, AB _m_AA _f_ and AB _m_BB _f_. Peaks near both extremes of 0% MAF exhibit a higher level of background noise and slightly differ from theoretically expected values. There are also some SNPs with MAF of 15–40% which are quite unlikely in theory. Existence of these polymorphisms may be explained by structural variations of genomic region or by uneven amplification favoring certain alleles over their counterparts, which may be very pronounced in highly polymorphic regions with tens of SNPs per amplicon. The latter hypothesis was confirmed after closer examination of unlikely polymorphisms: whereas in every sample they came typically from the same region, these regions did not replicate between different samples.

Large numbers of SNPs tend to have MAF near 0% and may be explained by sequencing errors. Those with an alternative allele percentage of 100% are probably sites where both the mother and fetus have the same genotype.

Some samples did not exhibit peaks at all ( Figure 5.C), probably because of absence of SNPs in required genotype configurations, which is expected from the fact that method doesn't require prior knowledge of parents' genotypes and instead relies on SNPs with high penetrance in the population. Introduction of additional regions and polymorphisms in them will overcome this issue.

High levels of background noise near 0% MAF prompted exclusion of these peaks, and further analysis was performed only on central ones. Concentration of cffDNA was calculated for 15 samples. Resulting values did not show correlation with the reference results (r=0.11, Figure 5.B). This fact suggests the need for method enhancement, primarily via a) using larger numbers of examined polymorphisms and regions, and b) elimination of PCR-introduced allele bias. Further we describe one approach to achieve this.

### cffDNA fraction estimation (deep sequencing of polymorphic regions excluding PCR-duplicates)

**Figure 6.**
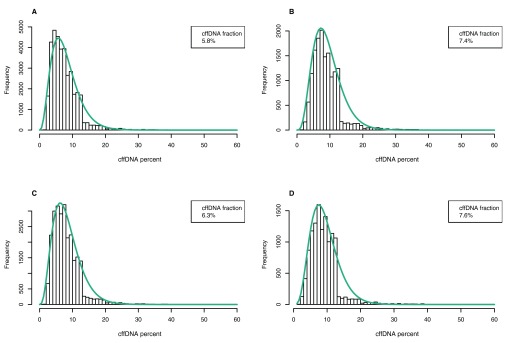
Distributions of cffDNA fractions calculated from allele frequencies of polymorphisms in different samples.

Another batch of samples was subjected to the modified approach. We employed an additional experimental technique to mark sequences originating from the same molecule during PCR, and changed the algorithm to calculate the cffDNA fraction separately for every found polymorphism; overall sample fraction would be the average of these. Per-SNP cffDNA fractions formed a gamma distribution, examples are presented in Figure 6. With these modifications in action, correlation with reference results reached 0.74. This marks the discussed method as a viable choice for further study in a sufficiently big sample cohort to allow it later for a commercial application.

## Conclusion

One of the aims of the current study was to assess the precision of our specific method in comparison to other NIPT techniques ^16– 18^. Sensitivity and specificity values obtained with the method regarding trisomy of chromosome 21 (95.6% and 99.8%, respectively) are in a good concordance with those of other reported NIPT practices. Determination of sensitivity and specificity for the 18th and 13th chromosomes will require bigger sample size due to small numbers of detected trisomies (4 and 1, respectively) and their lower incidence in general.

As expected, aneuploidies occurred more often in pregnancies of older women: 21 cases in a group of 35 years and older compared to 9 cases in those younger than 35 years. In the latter group 4 cases already had biochemical and/or ultrasonographic indications, and the other 5 had results of primary screening unknown. A group of younger women without any biochemical indications of elevated risk did not yield NIPT risk as well. On the contrary, in a group of women older than 35 years aneuploidies were detected in all subgroups, even in samples without biochemical or ultrasonographic risk indications.

We have shown that introducing an additional experimental step to eliminate PCR duplicates greatly enhances the precision of determining cffDNA concentration. Here it was implemented using the commercially available general purpose panel, yet further focusing only on relatively small number of polymorphisms with high MAF would be preferential due to lower coverage requirements and therefore lower sequencing costs.

Detection of aneuploidies through the estimation of cffDNA fraction shift between the chromosome in question and control autosomes shows some potential, but the statistical power of its current implementation might raise concerns enough to delay its in-field deployment. We propose further inquiry into the underlying mathematical models and expansion of sample sizes.

## Consent

Written informed consent for publication of their clinical details and/or clinical images was obtained from the patient/parent/guardian/relative of the patient. The study design was approved by the Institutional Review Board of Genoanalytica, CJSC, approval no. 103/2015.

## Data availability

The data referenced by this article are under copyright with the following copyright statement: Copyright: © 2016 Pantiukh KS et al.

Data associated with the article are available under the terms of the Creative Commons Zero "No rights reserved" data waiver (CC0 1.0 Public domain dedication). 

*F1000Research*: Dataset 1. Raw data for 'Report on noninvasive prenatal testing: classical and alternative approaches’, Pantiukh *et al.* 2015, 10.5256/f1000research.8243.d118686 ^19^

The raw sequencing data are available at the NCBI Sequence Read Archive ( http://www.ncbi.nlm.nih.gov/sra), accession number SRP072416.
