# Supplementary material for: Report on noninvasive prenatal testing: classical and alternative approaches
Source: F1000Res. 2016 Apr 22;5:722. [Version 1] doi: 10.12688/f1000research.8243.1 (PMC5054814; doi:10.12688/f1000research.8243.1)
Supplement: Peer review history

# Peer review: Referee response for version 1

**DOI:** 10.5256/f1000research.8865.r16320

**Published:** 2016-10-04

**Authors:** Sergey Nikolaev

**Affiliations:** 1 Department of Genetic Medicine and Development, University of Geneva, Geneva, Switzerland

The paper by Pantiukh et al. reports the results of the two novel NIPT approaches for the detection of aneuploidies.

 The work is performed on a large cohort of 1012 patients, all using appropriate controls. One of the methods under evaluation employs differential methylation status between cell free DNA in maternal blood of maternal and fetal origin. It is a very interesting idea to use this phenomenon for NIPT, which may have led to cost effective and fast diagnosis but unfortunately did not render promising results.

 However the other method, which is based on the assessment of VAFs of 220 common polymorphisms, is probably more promising. The presented results reached the correlation of 0.74 with the reference, which is still quite modest for the standards of diagnostics, but has potential for improvement with the increase of the number of tested SNPs. Among the disadvantages of this technique, which may potentially preclude it from wide use, is the utilization of sequence capture step which is time and labor consuming. Moreover, precap-PCR may bias the VAF distribution.

I have read this submission. I believe that I have an appropriate level of expertise to confirm that it is of an acceptable scientific standard.

# Peer review: Referee response for version 1

**DOI:** 10.5256/f1000research.8865.r13521

**Published:** 2016-06-13

**Authors:** Fyodor D Urnov

**Affiliations:** 1 Department of Molecular and Cell Biology, University of California Berkeley, Berkeley, CA, USA

The well-established positive correlation between maternal age and the incidence of newborns bearing chromosomal trisomies, combined with the decision that many women make to bear children later in life, makes prenatal genetic diagnostics both a field of major public health impact, and of relevance to individual women making reproductive choices. This places a significant burden of responsibility on the molecular geneticists developing such diagnostic methods to ensure their ease, scalability, sensitivity (ability to detect an aneuploidy), and specificity (not raising a false alarm). In this field, therefore, the excellent truly is the enemy of the good. Prenatal genetic testing, traditionally performed by invasive techniques, has in the past decade been replaced -- as a first-pass diagnostic -- with non-invasive genetic testing (NIPT), which is based on (1) the presence in maternal circulation of DNA of fetal origin and (2) the ability to use deep sequencing to karyotype the fetus at the molecular level. This approach has been reduced to routine clinical practice but, as is always the case, there is room for improvement. Pantiukh and colleagues present their findings on over 1,000 pregnancies in which both prenatal molecular analysis and newborn karyotype status are available, thus allowing an evaluation of novel methods for fetal karyotyping. The application of whole-genome low-coverage deep sequencing yielded data with clinical-grade sensitivity and specificity. Several additional approaches were evaluated, with variable, but in all cases informative, levels of success. In particular, an attempt to use epigenome profiling based on measuring DNA methylation levels, failed to provide robustly predictive results because of both experimental noise and variability in methylation itself. In contrast, an approach that uses SNP-based "counting" of alleles, while in need of further development, clearly shows potential. Overall the data in the paper give one confidence that building on existing approaches, in combination with novel ways to analyze fetal DNA, will continue to provide women with an ever-improving array of ways to make informed decisions about their reproductive choices.

I have read this submission. I believe that I have an appropriate level of expertise to confirm that it is of an acceptable scientific standard.
